# Editorial: Computational drug discovery for targeting of protein-protein interfaces—Volume II

**DOI:** 10.3389/fchem.2023.1171597

**Published:** 2023-03-07

**Authors:** Giulia Morra, Massimiliano Meli, Elisabetta Moroni, Alessandro Pandini

**Affiliations:** ^1^ Department of Chemical Sciences and Materials Technologies, Giulio Natta Institute of Chemical Sciences and Technologies, National Research Council (CNR), Milano, Lombardy, Italy; ^2^ Department of Physiology and Biophysics, Weill Cornell Medicine, Cornell University, New York, NY, United States; ^3^ Department of Computer Science, Brunel University London, Uxbridge, United Kingdom

**Keywords:** protein-protein interface (PPI), drug discovery, theoretical approach, computational methods, computational-experimental integration

Protein-protein and protein-peptide recognition are among the most important processes that make signal transmission and transduction possible in a cell; they also play a role in cellular processes involving diseases. Theoretical approaches in the study of these processes from a structural and thermodynamic viewpoint have focused on computational methods aimed at modelling, predicting, and characterizing the interface between the interacting proteins. Indeed, computational techniques represent potent tools for understanding PPI mechanisms at the atomic level: this information can then be exploited for designing molecules able to interfere with them.

However, designing molecules to directly targeting protein-protein interactions (PPI) is a difficult task. Differently from other computationally driven small molecule discovery efforts, PPI targeting presents unique challenges: high resolution structural information on targets is not easily available or predictable; PPI surfaces deviate from the conventional druggable sites targeted by therapeutic small molecules, being more hydrophobic, shallow and larger, thus having higher degree of conformational freedom. The unique nature of PPI is also reflected by known PPI inhibitors, which tend to have different physicochemical features from typical small compound drugs.

In this Research Topic, a few approaches are presented that try to overcome the complexity of designing PPI inhibitors or modulators by providing new computational methods or analysis tools to assist PPI drug discovery, as well as facilitate integration with experimental data.

Currently, no computational method can reliably predict the three-dimensional structure of protein complexes. Molecular docking is still an open challenge for protein-protein interactions, with one of the critical steps being the post-docking processing for determining the correct binding modes among the different models. Ranaudo et al. addressed this problem for affitins, a class of easily engineerable proteins that can be used as molecular probes able to specifically recognize biomarkers, for the diagnosis of serious diseases. In their work, they proposed a protocol for discriminating the correct from incorrect predictions of binding poses of different affitins in complex with several protein partners, obtained through an initial stage of rigid docking calculations. They demonstrated that a consensus approach based on Molecular Dynamics simulations and the prediction of potential interacting residues, through the Matrix of Local Coupling Energies method, leads to the identification of the correct binding pose. Their approach represents an effective tool for engineering affitins which are specific for different protein targets and, therefore, that could be used for diagnosis applications.

Catalytic activity in proteins is often allosterically regulated. He et al. studied an oncogenic system for which regulation is mediated by protein-protein interactions in a ternary complex. They explored the molecular and atomistic details of long-range allosteric effects in the G13D oncogenic mutant of K-Ras4B in complex with Son of Sevenless (SOS) protein. Their results suggest that in the ternary complex K-Ras4B^G13D^•SOS^cat^•K-Ras4B^G13D^–GTP, a GTP-bound version of Ras can allosterically activate the distal Ras bound to SOS. The authors used a set of well-established computational methods including network analysis to investigate conformational changes associated with the long-range allosteric mechanism. The novel insights provided by this study contribute to the current efforts to understand allosteric regulation in oncogenic protein complexes with the goal of informing drug development of inhibitors and modulators of protein-protein interaction.

The advanced application of drug discovery methods, among which of AI-assisted approaches, critically relies on the availability of annotated databases of compounds with given features such as chemical structures and physicochemical properties to drive the design of new hits. This is particularly true for the challenges posed by designing inhibitors targeting protein-protein interfaces, which typically deviate from the common features of small molecule inhibitors, e.g., in terms of molecular weight. Ikeda et al. present a novel chemical library database system (DLiP) to design PPI inhibitors, consisting of more than 32,000 PPI-related compounds. Among them, a significant component derives from *in silico* screening of a diverse set of protein-protein interfaces, through computational docking, while the rest is a Research Topic of experimental information from the literature or retrieved from other databases. Coming with a web interface that currently allows for several analyses and search, it promises to provide users with a download tool to be used in the future for prediction models.

Finally, an application stresses the importance of experimental validation of interaction predictions in the context of a pharmacologically fundamental problem. The protein-protein interaction lies at the heart of cell adhesion and the aberrant behavior of this mechanism is responsible for the spread of cancer cells and metastasis formation. As discussed in the paper by Vasile et al. the transmembrane protein E-cadherin is the principal actor in inter-cell adhesion and its downregulation gives the possibility for cancer cells to spread across the body and form metastasis. The formation of the complex between the extracellular moieties of E-cadherin could be a good target to prevent the reassociation of cancer cells. Towards this aim, the research of a new class of molecules able to interfere with E-cadherin homophilic interaction was carried out by combining *in silico* and experimental techniques. The *in silico* study was conducted with an initial analysis of the cavities and fragment-based drug discovery (FBDD). This technique allows us to identify which class of chemical fragment have a good chance to establish stable and strong interaction with the amino acids in the target protein. The Saturation-Transfer Difference Nuclear Magnetic Resonance (STD-NMR) experiments were used to validate the result obtained during the *in silico* simulations. With this experimental technique, it is possible to identify which part of the molecule interacts with the target protein. This research lays the foundation for further investigations towards the discovery of potent inhibitors.

Overall, this Research Topic presents a variety of problems and approaches, demonstrating a lively research area: the strategies and concepts suggested herein are likely to prove precious in increasing the possibility of generating new chemical tools useful in the biomedical and therapeutic fields. The increase in computational power and the development of new algorithms and computational strategies has significantly improved our understanding of experimental data on PPI targeting. In turn, experimental techniques have provided novel stimuli for the development and improvement of computational methods focused on the challenges of PPI targeting.

